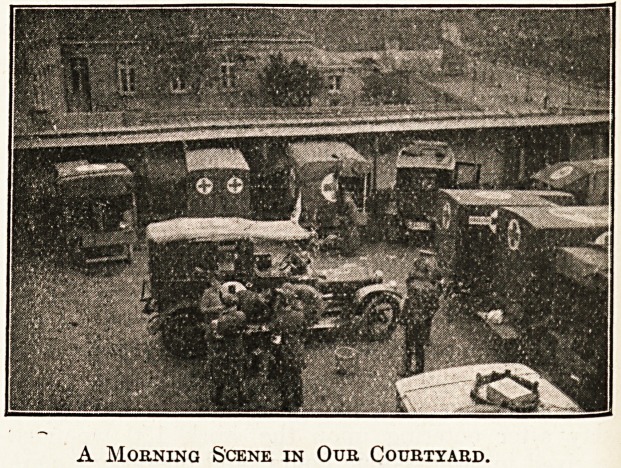# Voluntary Hospitals and Military Hygiene

**Published:** 1915-07-03

**Authors:** H. S. Souttar


### VOLUNTARY HOSPITALS AND MILITARY HYGIENE.

By H. S. SOUTTAR, F.R.C.S.

The second of the series of public lectures on this subject, under the auspices of the Chadwick Trust, was delivered by Mr. H. S. Souttar, F.R.C.S. (late Surgeon-in-Chief of the Belgian Field Hospital), who was supported by the presence of the late Belgian Minister in London, Count de Lalaing, at the House of the Royal Society of Medicine, on Thursday, the 24th ult.

Mr. Souttar said the Belgian Field Hospital was formed early last September as a purely voluntary organisation; a few people decided to try to go over to help the Belgians, and the suggestion was received with enthusiasm by the Belgian people. The telegram from the Queen of the Belgians accepting the services of the hospital proved an Open Sesame to every official, and nothing could exceed the cordiality of their welcome everywhere. On arriving at Antwerp, they were at once supplied with a completely equipped hospital containing 150 beds, a thoroughly equipped operating theatre, and extensive accommodation for the staff. On the fourth day after entering upon the work 150 wounded arrived in twenty-four hours, and operations were continuously going forward on two tables all day and the whole of the night, with very satisfactory results. Parties were sent to the trenches to assist the wounded there, but they did not bring the wounded from the trenches to the hospital; the distance was considerable, and if all had been brought in separately it would have disorganised the medical services. The patients were

Supper at Furnes, Belgian Field Hospital.

collected at certain dressing stations at the Front, and the staff of the hospital were allowed to bring their patients from the trenches to the dressing stations, and from the latter they were conveyed to the central station at Antwerp, whence they were allotted to the different hospitals in that city. The hospital cars were allowed to go anywhere, because the staff of the hospital only allowed those to go to the trenches who were likely to be of service, and who had a proper equipment for the wounded. With the growing pressure from the German army the work of the hospital proportionately increased.

The destruction and burning of a town like Termonde the lecturer described as a purely gratuitous

Our Chef.
A Wounded Belgian Soldier, in Civil Life Assistant Chef of Métropole, Brussels.

act; it contained no fortifications, and he thought there had been no pretence that the enemy were attacked by civilians there. The burning had obviously been in obedience to a settled plan, for each soldier was provided with inflammatory discs, which were set alight and thrown into the houses. Seventy British soldiers passed through the hands of the staff in four days, some of the wounds being very severe. He gave a vivid description of the bombardment of Antwerp, which began on October 7. A large shell reached the city about every five seconds, and when, on the following day, the evacuation of the hospital was ordered, there were 113 wounded patients, who had to be brought down the staircase into the basement prior to removal. This transfer to the basement was carried out in an hour without hurry or confusion, despite the fact that a shell might at any moment put an end to the process. The habit of the Germans seemed to have been to fire three shells at each point, the first two destructive and the third incendiary. It was a terrible journey through the night to Ghent. Eventually, after many vicissitudes, the hospital was established at Furnes, fifteen miles east of Dunkirk, just inside the circle of the German line, and so in a position to be of great service. The wounded were now generally in the hospital within six hours of the receipt of their injury in the trenches. He could not speak sufficiently highly of the staff of twenty-two fully-trained nurses. Though there were only 100 beds in the hospital no case was refused; a ward was laid thickly with straw, and on this many were accommodated and treated.

One of the great problems was as to what to do with the children they found; there were thirty or forty of them in a small street in Ypres the day following a heavy shelling of the town. Twelve were taken away and sent into France; but it was very difficult to know whether that was doing the best for them, and it could not be foreseen whether they were likely to see their parents again, so that their position was one of jeopardy. Indeed, the real tragedy of Belgium was not so much the wholesale destruction of buildings and property, sad as that was, but the destruction of the family life and the parting of relatives. The original photographs of the scenes described were very memorable and heart-rending, and formed a remarkable setting for the eloquent description of the work done by this beneficent agency.

A resolution of cordial thanks to the lecturer, carried by acclamation, was proposed by the Count de Lalaing, who said how deeply interested he was in the episodes of the journey of the hospital through Belgium, a most pathetic record which he did not like to dwell upon, though he knew of the intense sympathy of the English people for the country, which had been shown in so many ways. [The concluding lecture was delivered as we went to press.]

A Straw Ward.
(For Overflow.)

A Morning Scene in Our Courtyard.

## Figures and Tables

**Figure f1:**
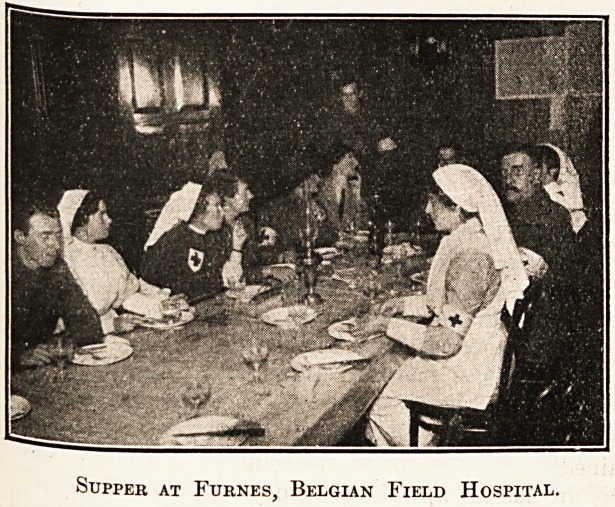


**Figure f2:**
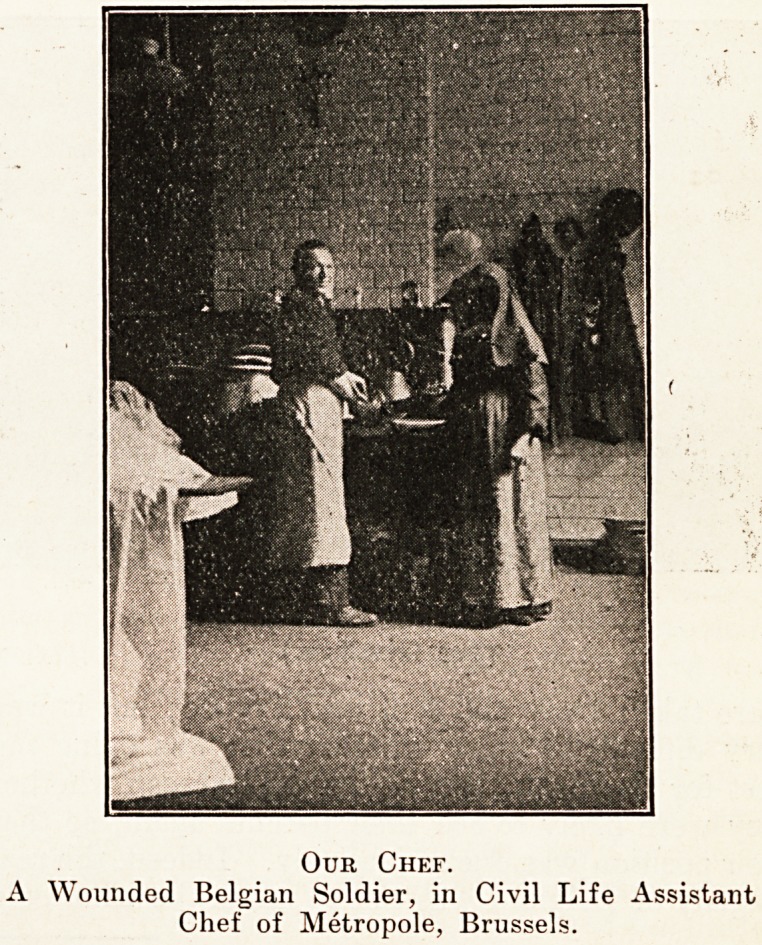


**Figure f3:**
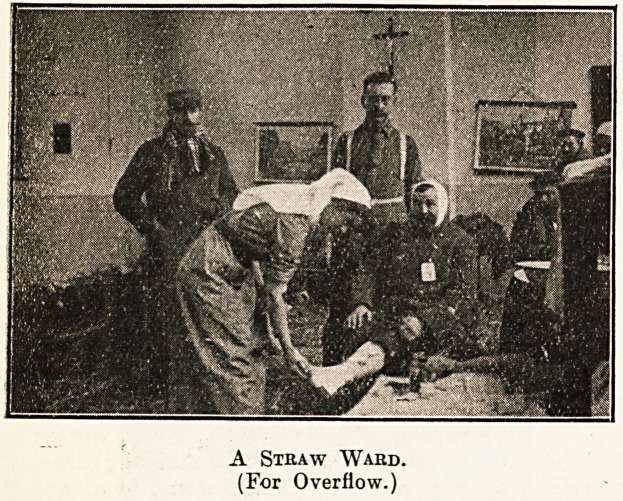


**Figure f4:**